# Supplementary material for: Collaborative intelligence in AI: Evaluating the performance of a council of AIs on the USMLE
Source: PLOS Digit Health. 2025 Oct 9;4(10):e0000787. doi: 10.1371/journal.pdig.0000787 (PMC12510544; doi:10.1371/journal.pdig.0000787)
Supplement: S2 File — (ZIP) [file pdig.0000787.s002.zip › S2 Data Raw data files containing unprocessed question inputs and LLM outputs including transcripts of deliberations/STEP3_Question-51-1_Type-MC-NJ.docx]

**Facilitator** **[Round 1]:** A 24-year-old woman, gravida 1, para 1, comes to the office because she and her husband have been trying to conceive for the past year without success. She gave birth to her first child at age 18 years by cesarean delivery following fetal distress. She then developed postpartum endometritis that resolved with intravenous antibiotic therapy. She used an intrauterine device (IUD) for 1 year following her first pregnancy but had the device removed because of intermenstrual bleeding. Medical history is also remarkable for chlamydial cervicitis treated with antibiotic therapy as an outpatient at age 21 years, and recurrent bacterial vaginosis that had been treated with metronidazole therapy during her pregnancy and on two other occasions. Vital signs today are within normal limits. Breast and pelvic examinations disclose no abnormalities. Hysterosalpingography shows bilateral hydrosalpinx. Which of the following factors in this patient's history most increased her risk for development of her condition? (A) Bacterial vaginosis (B) Cesarean delivery (C) Chlamydial cervicitis (D) Postpartum endometritis (E) Use of an IUD

**AI_D:** RESTATING THE QUESTION: In essence, we are trying to understand the key factor in a 24-year-old woman's medical history, having one pregnancy in her past, that has contributed the most to her current condition of bilateral hydrosalpinx. Bilateral hydrosalpinx refers to a condition where both Fallopian tubes encounter an obstruction filled with serous or clear fluid - this condition is commonly associated with infertility. The potential contributors to this woman's condition include bacterial vaginosis (a mild infection of the vagina due to an imbalance of bacteria), cesarean delivery (a surgical delivery procedure), chlamydial cervicitis (inflammation of the cervix due to a sexually transmitted infection), postpartum endometritis (inflammation of the uterine lining following childbirth), and the use of an IUD (a device inserted into the uterus to prevent pregnancy). PLANNING: Certainly, each of the propositions can contribute to the cause of hydrosalpinx, as inflammation or infection of any part of the uterus, cervix, and vagina can potentially lead to the Fallopian tubes. However, the one condition that most often results in hydrosalpinx is pelvic inflammatory disease, primarily caused by sexually transmitted infections, with Chlamydia trachomatis being the most common infecting organism. CLINICAL REASONING: Connecting the dots, chlamydial cervicitis indicates an infection with Chlamydia trachomatis, a sexually transmitted bacterium that can ascend the genital tract and cause pelvic inflammatory disease. Pelvic inflammatory disease can lead to long-term sequelae including chronic pelvic pain, ectopic pregnancy, and infertility secondary to tubal obstruction, such as hydrosalpinx. While the other factors in the patient's history may be contributory, chlamydial cervicitis presents the most plausible and direct path to the development of the patient's hydrosalpinx. Bacterial vaginosis and use of an IUD, for instance, can increase the risk of pelvic inflammatory disease, but these are more generalized risks. CONCLUDING: The chronology of this patient's medical history, combined with the known complications of chlamydial cervicitis, makes it a predominant factor in contributing to her current condition of bilateral hydrosalpinx, and as a result, her infertility. RESPONSE: So, of the options provided, (C) Chlamydial cervicitis most significantly increased this patient's risk for developing her current condition.

**AI_A:** 1) RESTATING THE QUESTION: The inquiry focuses on determining which single factor from this 24-year-old woman's medical history has primarily contributed to her current condition of bilateral hydrosalpinx, significantly reducing her fertility. The suspected risk factors include bacterial vaginosis, cesarean delivery, chlamydial cervicitis, postpartum endometritis, and use of IUD. Hydrosalpinx refers to a condition where the fallopian tubes fill up with fluid due to inflammation or infection, which can lead to female infertility. 2) PLANNING: The cited risk factors all entail various impacts on women's reproductive health. Bacterial vaginosis could lead to pelvic inflammatory disease (PID), but is generally less associated with tubal damage compared to chlamydial infection. Cesarean delivery remains unlinked with fallopian tube damage. Chlamydial cervicitis, a sexually transmitted infection, significantly increases the risk of PID, subsequently leading to tubal damages like hydrosalpinx. Postpartum endometritis occurs due to a uterine infection following delivery or miscarriage; despite being severe, it isn't linked to tubal pathology. IUD can potentially contribute to PID which subsequently could lead to tubal damages in rare cases. It is pertinent to evaluate each of these assumptions in the given clinical context. 3) CLINICAL REASONING: Bacterial vaginosis, while potentially leading to PID, is less likely the primary risk factor as chlamydial infection is known with more relative certainty to cause PID and subsequent fallopian tube damage. Cesarean delivery is not typically linked to hydrosalpinx. Postpartum endometritis, although a severe condition, doesn't normally lead to damage of the fallopian tubes. Use of an IUD could contribute to the development of PID and subsequent tubal damage, though this tends to be rare and with the observation of recurrent chlamydial cervicitis, the contribution from IUD is likely lesser. 4) CONCLUDING: Considering the woman has a history of chlamydial cervicitis, a known risk factor for PID leading to tubal inflammation and potential damage, it is plausible to assume that this could have contributed to her bilateral hydrosalpinx, consequently impairing her fertility. This assumption aligns with the patient's overall clinical presentation and her fertility problems as a consequence of the hydrosalpinx which resulted from inflammation or infection of the fallopian tubes. 5) RESPONSE: Therefore, in this patient's case, the factor that most likely increased her risk for the development of her current condition, bilateral hydrosalpinx, is (C) Chlamydial cervicitis.

**AI_E:** RESTATING THE QUESTION: A 24-year-old woman who has had one successful pregnancy but is struggling to conceive again presents with a medical history of multiple infections - postpartum endometritis, chlamydial cervicitis and repeated bacterial vaginosis. She's also had intrapartum complications in her first pregnancy, resulting in a cesarean delivery and a history of intrauterine device usage. Current diagnostic evaluations reveal bilateral hydrosalpinx( dilated and fluid-filled fallopian tubes). Identifying which event had the highest propensity to cause her current complications is the query. PLANNING: Assumptions that need to be considered are whether one or multiple factors have contributed to her current condition. Each factor suggested - bacterial vaginosis, cesarean delivery(wherein an incision is made through the mother's abdomen and uterus to deliver one or more babies), chlamydial cervicitis(inflammation of the cervix caused by Chlamydia trachomatis), postpartum endometritis(inflammation or infection of the uterus after childbirth) and use of an IUD can individually lead to problems in conception. While these all have potential links to infertility, one needs further examination in the context of her observed condition - bilateral hydrosalpinx. CLINICAL REASONING: Hydrosalpinx, or the dilation of the fallopian tubes filled with fluid, can occur as a result of scarring from past infections that have caused damage to the delicate tubal structures. Infections capable of causing this include sexually transmitted diseases, such as Chlamydia (C. trachomatis) - the pathogen responsible for chlamydial cervicitis. However, in broader considerations, postpartum endometritis could also have led to tubal damage if the infection ascended from the uterus to the fallopian tubes, although this is statistically less common. Bacterial vaginosis, while also infection-related, commonly does not result in hydrosalpinx. IUD use and cesarean delivery seldom lead to this specific pathology. CONCLUDING: Based on the clinical relevance and the higher epidemiological incidence of pelvic inflammatory disease as a result of C. trachomatis infection, it is arguable that her chlamydial cervicitis experience at age 21 years stands as the most likely major contributor to her present hydrosalpinx condition, which could explain the inability to conceive. RESPONSE: Hence, my answer according to the provided choices would be (C) Chlamydial cervicitis.

**AI_C:** (1) RESTATING THE QUESTION: This is a question about a 24-year-old woman who has been trying to conceive for the past year without success. Her medical history shows a cesarean delivery, postpartum endometritis, use of an IUD, chlamydial cervicitis, and recurrent bacterial vaginosis. Her recent Hysterosalpingography revealed bilateral hydrosalpinx. We need to understand which of these factors might have contributed to her inability to conceive and led to her current condition. (2) PLANNING: Bilateral hydrosalpinx refers to a blockage in both fallopian tubes where they attach to the uterus, causing their dilation and filling with fluid. Several assumptions could be made: the postpartum endometritis, chlamydial cervicitis, or the recurrent bacterial vaginosis could be risk factors for pelvic inflammatory disease, which could have damaged her tubes. Or, the use of IUD might have caused tubal damage, or the cesarean delivery could have led to adhesions and blockage. However, chlamydial infections are known to present more often as silent infections, slowly causing tubal damage without overt symptoms. Let's explore this assumption further. (3) CLINICAL REASONING: Postpartum endometritis is characterized by an infection of the uterine lining following childbirth, which could cause scarring but is less likely to directly affect the fallopian tubes. IUDs may cause increased risk of PID, primarily if there is an existing STD, but they do not generally trigger tubal damage in sterile conditions. A C-section could cause adhesions in the uterus and nearby structures, but these are most likely located at the site of the scarring, not extending to the distal fallopian tubes. Bacterial vaginosis is a vaginal condition that can lead to PID, but only if it ascends up into the uterus and fallopian tubes. On the other hand, chlamydia, a sexually transmitted disease, specifically infects the cervix and can ascend to the uterine tubes, causing inflammation, scarring, and blockage - exactly like hydrosalpinx. (4) CONCLUDING: Given the clinical characteristics of the medical history provided and the contribution to tubal damage, we can conclude that the factor that most increased the risk for the development of this patient's hydrosalpinx condition is her history of chlamydial cervicitis. (5) RESPONSE: Therefore, based on the woman's medical history and current condition, the answer is (C) Chlamydial cervicitis.

**AI_B:** RESTATING THE QUESTION: We need to determine the major contributing factor in this patient's history that has led to her current condition: bilateral hydrosalpinx, demonstrated by a hysterosalpingogram. To make this determination, we have to evaluate how her prior medical history, including bacterial vaginosis, cesarean delivery, chlamydial cervicitis, postpartum endometritis, and the use of an IUD, could have influenced her current condition. PLANNING: To determine the highest contributing factor, we need to consider each presented condition independently and see how they could contribute to the development of hydrosalpinx, a condition characterized by fluid accumulation in the fallopian tubes, often due to inflammation or infections. CLINICAL REASONING: Let's consider each potential contributor. (A) Bacterial Vaginosis: While this can lead to pelvic inflammatory disease, and subsequently result in hydrosalpinx, the recurrent but successfully treated nature of her bacterial vaginosis makes it an unlikely primary cause. (B) Cesarean delivery: A C-section can lead to adhesions but isn't commonly associated with bilateral hydrosalpinx. (C) Chlamydial Cervicitis: Chlamydia infection can lead to pelvic inflammatory disease if untreated, and chronic infection can cause hydrosalpinx. This is a potential contributor. (D) Postpartum Endometritis: Inflammation of the uterine lining can lead to scarring and inflammation spreading to the tubes, possibly contributing to hydrosalpinx. (E) Use of an IUD: An IUD is associated with an increased risk of pelvic infection, but not typically associated with hydrosalpinx. CONCLUDING: Considering the prior medical history, although each condition could potentially enhance the risk of hydrosalpinx, untreated and chronic chlamydial cervicitis generally contributes to pelvic inflammatory disease and eventually the blockage of fallopian tubes more than other conditions listed. RESPONSE: The factor that most increased her risk for development of bilateral hydrosalpinx in her medical history is (C) Chlamydial cervicitis.

**Facilitator** The consensus among the AI responses is that the primary factor in the 24-year-old woman's medical history that most likely contributed to her current condition of bilateral hydrosalpinx, and thus her fertility issues, is her history of chlamydial cervicitis. This conclusion is based on the understanding that chlamydial cervicitis, caused by the sexually transmitted bacterium Chlamydia trachomatis, can ascend the genital tract and cause pelvic inflammatory disease. This disease can lead to long-term complications including chronic pelvic pain, ectopic pregnancy, and infertility secondary to tubal obstruction, such as hydrosalpinx. While the other factors in the patient's history, including bacterial vaginosis, cesarean delivery, postpartum endometritis, and the use of an IUD, may have contributed to her condition, chlamydial cervicitis presents the most plausible and direct path to the development of the patient's hydrosalpinx. Therefore, the answer to the question is (C) Chlamydial cervicitis.
